# Contemporary Theory and Research

**Published:** 1893-04-22

**Authors:** 


### Contemporary Theory and Research.

### A SIMPLE REMEDY FOR BURNS.

M. Bienert strongly advocates the use of glycerine for wounds caused by burns or scalds. Poured immediately on the parts affected or used in bandages it speedily soothes the pain, and except for a slight redness there remains the day after a burn neither swelling nor pain. Glycerine, he maintains, is superior to all other remedies commonly employed.

### A REMEDY FOR STERTOROUS BREATHING.

Dr. Rand contributes the following to the *New York Medical Record*: "On Friday February 10th, I called upon an old man dying of apoplexy. As soon as I entered the house, I noticed his harsh, heavy breathing, although the room that he occupied was beyond that adjoining the hall. The nurse stated that this had been the character of his respiration for seven hours, despite all attempts to relieve him by frequent changes of position. The mouth was opened and of course dry, the tongue drawn far back, and the lips fallen in. As I watched him the thought of opening the glottis by placing the fingers behind the angles of the lower jaw and bringing it forward, as we do when respiration becomes embarrassed in surgical anæsthesia, occurred to me. I did this and relief was instantaneous. He breathed as noiselessly as a child. By a bit of experimenting I found that pressure upward and forward under the chin produced the same result. So protecting the flesh with a hankerchief, a cardboard prop from the chest was improvised. With little care on the part of the nurse this was kept in position, and from that time throughout the twelve hours which the patient survived, the respiration continued quiet and natural. I am sure that occasionally cases are seen in which the remedy named will afford more real relief both to patients and attendants than everything else that can be done by the physician."

### THE COMPLICATIONS OF INFLUENZA.

M. Marvand, in a work on the influenza epidemic in the Lyons garrison, cites several curious cases of cutaneous eruptions among influenza patients which occasioned some doubt about the diagnosis. Certain patients showed every symptom at the commencement of an attack of measles; in six patients the rash had all the appearance of scarlatina; yet all these cases proved to be genuine examples of "la grippe." It would seem there is hardly any disease with which this mysterious complaint has not some affinity.

### A DANGEROUS INDUSTRY.

It is not perhaps generally known under what hurtful conditions the culture of rice is carried on. It necessitates, in fact, the inundation of the tract of country where it is cultivated, and obliges the labourer to carry on his work during a portion of the year with his legs submerged in stagnant water. Accordingly, in the rice districts of Piedmont and elsewhere the population has hitherto been consumptive and decimated by disease. Dr. Tullio Bonizzardi has been calling attention to the excellent service lately rendered by the proprietor of the Vianna rice district in Italy in introducing on his property improvements which have completely put an end to the insanitary conditions formerly deemed inevitable to this industry. The levelling of the ground and the construction of irrigation works have transformed these marshy plains. A pure and limpid air has replaced the former thick and unhealthy fogs; the stagnant waters have given place to clear streams; health has replaced disease, and the excellent sanitary conditions of the colonists is reported.

